# Interventions to improve referrals from primary care to outpatient specialist services for chronic conditions: a systematic review and framework synthesis update

**DOI:** 10.1186/s13643-025-02841-z

**Published:** 2025-05-09

**Authors:** Layla Bolton Saghdaoui, Smaragda Lampridou, Sara Tavares, Rachael Lear, Alun Huw Davies, Mary Wells, Sarah Onida

**Affiliations:** 1 Section of Vascular Surgery, Department of Surgery and Cancer, Charing Cross Hospital, Imperial College London, Fulham Palace Rd, 4th Floor (North), Vascular Outpatients, Room 4N22C, London, W6 8RF UK; 2 Hanwell Health Centre, School of Public Health, Imperial College London, London, W7 1DR UK; 3 White City Campus, The George Institute for Global Health, Public Health School, Imperial College London, London, UK; 4 Digitial Collaboration Space, Imperial College London, 1A Sheldon Square, W2 6PY, Queen Elizabeth the Queen Mother Wing (QEQM), St Mary’s Campus, London, UK; 5 Faculty of Medicine, Education Centre, Charing Cross Hospital, Imperial College London, Fulham Palace Rd, London, W6 8RF UK

**Keywords:** Referral, Primary care, Specialist services, Chronic conditions

## Abstract

**Background:**

Prior systematic reviews highlight that accessing specialist healthcare to treat chronic conditions can be obstructed by variations in referral rates, inappropriate referrals, and poor communication. Structured referral proformas, peer feedback, and educational interventions involving specialists have been identified as successful strategies for improving referral rates and appropriateness. However, the success of such interventions is often dependent on specific clinical contexts, and little is known about the practicalities of implementation. Additionally, with advancements in healthcare delivery, such as e-referral systems, there is a need to explore new interventions and how they address barriers to referral.

**Methods:**

This systematic review evaluated the updated evidence exploring interventions aiming to improve rates and/or appropriateness of referral from primary care to specialist services in patients with chronic conditions.Five academic databases were searched (CINAHL, MEDLINE, Embase, British Nursing Index, and Public Health Database), and studies published in English between 2013 and 2023 were included. The Joanna Briggs Institute’s appraisal tool was used to assess the quality of studies, and a narrative synthesis was conducted using the TiDiER framework (template for intervention description and replication).

**Results:**

Eighteen full-text publications and five abstracts were included. A behavioral theory or framework for intervention development was used in seven studies. All interventions were based on primary care, and thirteen studies evaluated a multi-component intervention. Process and system changes were most commonly used to improve referral, including electronic health systems, referral algorithms, collaborative working, and patient direct access. Interventions targeted at patients were the least common. Staff education was often used in addition to process and system changes. When used alone, referral algorithms and staff education were less effective at improving referral rates or appropriateness. Implementation barriers included time constraints, logistical issues, and patients/staff preconceived perceptions of referral necessity.

**Conclusion:**

Unsurprisingly, the success of interventions aimed at improving referral practices is based on contextual circumstances, and as with previous reviews, there is no one-size-fits-all approach.Given the challenges highlighted in this review, multi-component interventions addressing referral barriers in both primary and secondary care appear to be a successful way to improve referral practices.

**Review registration:**

PROSPERO CRD42023480493

**Supplementary Information:**

The online version contains supplementary material available at 10.1186/s13643-025-02841-z.

## Background

Primary care services are often a patient’s main point of access to a healthcare system. With increasingly complex populations living with multiple long-term conditions, the World Health Organization describes such services as being optimally positioned to facilitate early diagnostic services, coordinate care pathways for chronic disease, and maintain overall health and well-being [1, 2]. While traditionally led by general practitioners (GPs), modern practices include a range of healthcare professionals such as nurse practitioners, pharmacists, and healthcare support staff, all of whom could be responsible for referring patients to specialist services [3, 4]. Specialist services can be situated in primary, secondary, and tertiary care and support those with more complex or rare conditions. Of 149 specialized services commissioned in the UK, most referrals in the National Health Service (NHS) are attributed to a chronic healthcare issue, with musculoskeletal problems, rehabilitation, wound and foot care, frailty, continence issues, and nutrition being the top reasons for referral in 2022 [5, 6].

Although referral has implications for both patients and healthcare systems, evidence across clinical specialties points towards unexplained variations in referral rates, inappropriate referrals, and poor communication between primary care and specialist services [7–9]. To address this, several interventions have been tested across different clinical specialties, leading to a number of systematic reviews evaluating referral processes and shared care [10–12]. Early reviews concluded that there was a lack of rigorous evaluation and high-quality evidence on how best to tackle this issue [11]. Moreover, passive dissemination of referral guidelines was found to be ineffective, whilst structured referral proformas and local educational interventions involving secondary care specialists were more successful at improving referral rates and appropriateness [11].

Following the results of the first published review [11] in 2014, a large-scale evaluation of international evidence was completed [10]. Evaluating 290 studies using a logic model, the authors organized interventions into four categories: GP education, process changes, system changes, and patient-focused interventions. Although peer feedback on the content of referrals reduced referral rejection rates, success was largely dependent on location and clinical context [10].

Evidence also suggests that adopting an approach targeted at both primary and secondary care is more effective than single-component interventions solely based in a primary care setting. Given this, it is unsurprising that many studies adopted a multi-component design targeting primary and specialist services [10, 13]. Similarly, another review concluded that multi-component interventions, incorporating approaches such as referral guidelines, templates, and healthcare provider peer feedback, effectively improved referral rates, the quality of the information provided in referrals, and staff satisfaction. While peer feedback was found to increase referral quality and decrease inappropriate referrals by up to 50%, the authors also stress that clinicians’ and patients’ perceptions, behavior, and attitudes regarding referral inevitably impact the applicability and effectiveness of interventions [12]. For example, the perception of referral quality sometimes differed between GPs and specialists. In one study, 69.3% of GPs felt they usually included all relevant clinical information. However, only 34.8% of specialists reported receiving the details they needed [14].

While evidence suggests that certain intervention methods may be effective, it also depicts a complex referral process dependent on clinical context and marked by fragmented roles between healthcare professionals [10]. At present, no recent reviews explicitly explore how interventions are developed, and it is unknown whether interventions are guided by behavioral theories that consider the impact of perceptions and behaviors on intervention effectiveness. This is significant as the UK Medical Research Council’s guidance on developing and implementing complex healthcare interventions [15] outlines the importance of such steps to ensure interventions are contextually sensitive, feasible, and pragmatic, allowing for seamless implementation into real-world clinical practice.

In addition to this gap in the current evidence, in the UK, there have been several developments in how we deliver healthcare services, including the implementation of the NHS e-referral service, replacing the choose and book system [16]. Consequently, there is a need for a comprehensive understanding of the updated literature on interventions aiming to improve the referral of patients from primary care to specialist services and an assessment of how they may be applicable in the context of the NHS.

### Research question

To explore what interventions may be applicable within the NHS, this review will update and extend the findings of Blank et al. by evaluating the following:

- What is the effectiveness of existing interventions in improving the rates and/or appropriateness of referral from primary care to outpatient specialist services in patients with chronic conditions?
- What barriers and facilitators exist when implementing interventions to improve rates and/or appropriateness of referral from primary care to outpatient specialist services in patients with chronic conditions?

### Aims of this review

1. To identify interventions and their components aimed at improving rates or appropriateness of referral from primary to secondary care
2. To identify mechanisms of referral, e.g., electronic, verbal, and post
3. To evaluate the effectiveness of interventions to improve referral from primary to secondary care outpatient services
4. To identify what factors affect the implementation of interventions aimed at improving rates or appropriateness of referral

## Methods

This review was conducted as an update of the work published by Blank et al. (What is the evidence on interventions to manage referral from primary to specialist non-emergency care? A systematic review and logic model synthesis, 2015.) [6] and was guided by the methods used in their publication. In doing so, a narrative synthesis approach was applied alongside the use of a logic model summary. Results have been reported in line with the Preferred Reporting Items for Systematic Reviews and Meta-Analyses (PRISMA) checklist [17]. Before undertaking this review, it was registered on the international database of prospectively registered systematic reviews (CRD42023480493).

A systematic search of the literature was conducted using the following databases: CINAHL (Ebsco), MEDLINE (Ovid), Embase (Ovid), British Nursing Index (Proquest), and Public Health Database (Proquest). Additionally, the platform Trip Pro Database was searched to identify any gray literature, such as local hospital reports, that may not have been captured in the original search. While searches were initially carried out in November 2023, all were updated in July 2024. Once studies were selected for inclusion, their reference lists were reviewed to ensure relevant papers had not been missed.

### Search strategy and study selection

The search terms used in previous reviews [10, 11] were adopted for this updated search. However, in earlier reviews [10, 11], specific terms such as “Gatekeeping,” “Clinical Competence,” and “doctor knowledge” were applied to increase search specificity [10, 11]. We decided not to use these terms to ensure no relevant research was excluded. A full list of search terms and the outline of searches for each database can be found in Supplementary file 1.

Study selection was conducted by authors LBS, SL, and ST. To do this, the online platform Covidence (https://www.covidence.org) was used to remove duplicates and carry out independent first-level screening and full-text review. Firstly, this included reviewing the titles and abstracts of all results in line with the inclusion and exclusion criteria. Similarly, full-text results were then independently reviewed by two authors. Any discrepancies were resolved by (MW and AHD).

### Inclusion and exclusion criteria

#### Types of study

Previous systematic reviews evaluating referral process interventions report study design heterogeneity [10, 11], so the following study designs were included: randomized controlled trials, observational studies (e.g., cross-sectional studies, cohort studies, case–control studies), and qualitative studies. Abstract publications were included if they described mechanisms of referral.

Only studies published in English were included due to the limitations on costs and resources available for translation. As this review is an update of Blank et al., [10] only studies from 2013 onwards were considered. To ensure the findings are transferable to the UK health system, only studies evaluating healthcare structures in developed countries (categorized as per the United Nations specifications) were included [18].

#### Population

Studies were included if they examined interventions to improve the referral of adult patients (> 18) from primary care to outpatient specialist services for specialist outpatient advice regarding a chronic condition. A chronic condition was defined as a health issue requiring ongoing medical attention [19]. All primary care healthcare professionals (HCP) with the ability to refer adult patients to a secondary care outpatient specialist service were included.

#### Types of intervention

Studies reviewing interventions aimed at the referral of patients with acute or life-threatening conditions were excluded. When interventions examined both chronic and acute patients, only studies that allowed disaggregation of data were considered. Although cancer can be considered a chronic condition, in the UK, patients with a suspected or confirmed cancer diagnosis follow a very specific referral pathway (often a two-week wait) [19]. Consequently, studies exploring the referral of cancer patients were excluded. Both single and multi-component interventions were included.

#### Intervention outcomes

Studies evaluating the following outcomes included referral rates, referral quality, appropriateness of referral, impact on existing service provision, costs, mortality and morbidity outcomes, length of stay in hospital and safety, quality of life, effectiveness, patient satisfaction, staff satisfaction, patient experience and process measures, and all qualitative outcomes.

#### Comparators

No limits were applied to the type of comparators used to evaluate an intervention. In previous reviews, the majority of studies either did not have a comparator or evaluated against current standard practice.

### Data extraction and data synthesis

Using the online platform Covidence (https://www.covidence.org), a data extraction form was developed and informed by the TiDiER framework (template for intervention description and replication) [20] that covers twelve required items outlined in Table 1*.* First-level data extraction included authors, study location, year of publication, study design/methods, data collection method, study aims, number of participants, participants characteristics, intervention details, control details, length of follow-up, response and/or attrition rate, context (referral from what/who to what/who). In keeping with the previous reviews [10, 11], all other outcome measures were extracted following this. Outcomes were recorded using the statistical outcome measures presented by the authors, such as relative risk, odds ratio, or *p*-value. Where authors cited a published protocol describing intervention development, use of behavioral theory, or use of an intervention framework, these papers were also used for data extraction. As with study selection, this process was conducted independently by two authors (LB and SR), and where discrepancies occurred, this was reviewed by a third author (ST).

**Table 1:** TiDiER framework

	**TiDiER framework**
Item 1	Name of intervention
Item 2	Rationale and theory for intervention
Item 3	Physical or informational materials required for the intervention
Item 4	Intervention procedures or activities
Item 5	Provider of intervention (e.g., nurse/doctor/researcher)
Item 6	Modes of delivery (e.g., face-to-face/telephone consultations)
Item 7	Location of intervention
Item 8	Delivery and time periods of intervention
Item 9	Tailoring of intervention (e.g., personalized, titrated, or adapted)
Item 10	Intervention modifications
Item 11	Intervention evaluation
Item 12	Intervention adherence

Due to the difference in study designs, outcomes measured, and intervention aims, a meta-analysis or aggregation was not possible. To guide data synthesis, the framework synthesis approach [21] was followed by adhering to five key stages: familiarization, framework selection, indexing, charting, and mapping. The first two stages include getting to know the empirical literature and selecting an initial framework, logic model, or established theory. In the indexing and charting stages, the framework is used to organize and extract data from included studies. It then develops iteratively as new data are incorporated and additional themes develop [21]. This approach has been chosen as the previous review [10] developed a logic model to illustrate the pathway from intervention to intended outcomes and includes the following five headings: intervention types, immediate effect, predictors of referral behavior (describing predictors of change, e.g., barriers or facilitators) and overall outcomes and impact. As per the logic model, interventions were categorized further into the following four groups: GP education, process changes, system changes, and patient-focused interventions. Similarly, immediate effect outcomes were categorized into the following groups: HCP knowledge, HCP attitudes and beliefs, referral behavior, HCP and patient interaction, patient knowledge, and patient attitudes and beliefs. While adopting this logic model included utilizing these headings for the data extraction template, it was equipped with an “other” tab, allowing the model to develop further when new information outside of its scope was identified.

### Quality appraisal

The Joanna Briggs Institute’s (JBI) critical appraisal tools were used to assess the quality of the studies according to their study design [22]. Publications detailing a mixed methods approach were appraised as such [22]. The JBI checklists require users to give a rating of “yes,” “no,” “unclear,” or “not applicable” to each question [22]. One point was given for every question answered as ‘yes.’ Following this, a total score was calculated for each study. This was done to evaluate the quality of the evidence currently available; however, following an inclusive approach, no studies were excluded based on quality assessment scores.

## Results

**Fig. 1:**
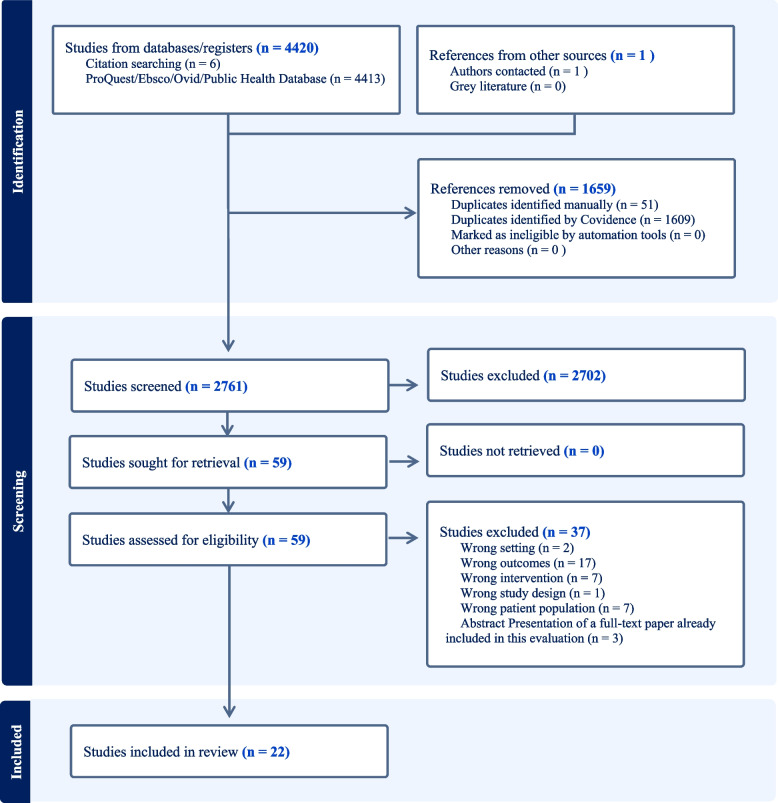
PRISMA diagram

The literature search in this updated review initially yielded 4421 publications. After the abstract and full-text screening process, 23 studies were evaluated, including six abstracts and seventeen full-text publications (Fig. 1). The lead authors for each identified abstract were contacted in an attempt to determine if the full study results had been published. Three abstracts had no listed contact details for the authors. One listed email was no longer receiving correspondence. One author was emailed but did not respond. One author was emailed and provided the full-text publication for the abstract. For this study both the abstract and full text paper have been included and fully study characteristics can be found in Table 2.

Of the full-text studies reviewed, seven achieved a score of 50% or more when appraised for quality, and eight scored below 50% (supplementary file 2, tables 1- 7). The five abstracts (not accompanied by a full test publication) identified were not included in the quality appraisal process due to the limited information provided on the methodological processes.

**Table 2:** Study characteristics

Type of publication	Lead author	Title	Country	Study design	Clinical specialty	Intervention type	Immediate effect	Predictors of referral behavior	Intervention outcomes/intended impact
Abstract	Bhamra, 2019	Food as medicine: medical nutrition therapy for patients living with diabetes	USA	Cross-sectional study	Diabetes	Process change System change	Referral behavior	Service factors	Referral rate
Abstract	Chow, 2015	A multidisciplinary effort to improve utilization of resources for patients with morbid obesity in the primary care clinic	USA	Quality improvement	Morbid obesity	HCP education System change Patient intervention	HCP knowledge Referral behavior	HCP factors Service factors	Referral rate Mortality and morbidity outcomes
Abstract	Lavenbur, 2023	Primary care provider perceptions of a population health management program to improve kidney care	USA	Qualitative research	Chronic kidney disease—nephrology	Process change	HCP attitudes and beliefs Referral behavior	HCP factors Service factors	Impact on existing service provision Staff satisfaction
Abstract	Liao, 2023	Can a Multidisciplinary Virtual Educational Seminar Change Resident Physician Attitudes and Referral Practices to Complementary and Integrative Health?	USA	Survey, qualitative research, and cohort study	Physiotherapy—low back pain	HCP education	HCP knowledge HCP attitudes and beliefs Referral behavior	HCP factors	Referral rate Staff satisfaction
Abstract	Shrivastava, 2018	An allied health professional-led annual health clinic to improve standards of care	UK	Cross-sectional study	HIV	Process change	Referral behavior	HCP factors Service factors	Referral rate Referral quality Appropriateness of referral
Abstract & full text	Filippini, 2014 and 2022	Implementation of an Integrated Care Pathway for dementia in the clinical practice: rationale, design, and methodology. The REMIND Study	Italy	Cohort study	Neurology—dementia decline detection	HCP education Process change	HCP knowledge HCP attitudes and beliefs Referral behavior	HCP factors Service factors	Referral rate Referral quality Appropriateness of referral
Full text	Barnett, 2015	Implementation of an electronic referral system in a large academic medical center	USA	Cohort study	Various clinical specialities	Process change System change	Referral behavior	HCP factors Service factors	Referral rate Referral quality Appropriateness of referral Staff satisfaction
Full text	Bishop, 2015	A pilot cluster randomized controlled trial to investigate the addition of direct access to physiotherapy to usual GP-led primary care for adults with musculoskeletal pain: the STEMS trial	UK	Pilot pragmatic, non-inferiority, cluster randomized controlled trial	Physiotherapy—musculoskeletal pain	Process change HCP education	HCP—patient interaction Patient attitudes and beliefs	Patient factors	Referral rate Referral quality Appropriateness of referral Impact on existing service provision Costs Quality of life Effectiveness Patient satisfaction
Full text	Disler, 2022	A new model for general practice-led, regional, community-based, memory clinics	Australia	Cross-sectional study	Dementia	HCP education Process change System change	Referral behavior	Service factors	Referral rate Referral quality
Full text	Haley, 2015	Improving care coordination between nephrology and primary care: a quality improvement initiative using the Renal Physicians Association toolkit	USA	Other: qualitative research/longitudinal cohort study	Nephrology	HCP education Process change	HCP knowledge HCP attitudes and beliefs Referral behavior	HCP factors Service factors	Referral rate Referral quality Appropriateness of referral
Full text	Jamal, 2020	The IMPACT study: a clustered randomized controlled trial to assess the effect of a referral algorithm for axial spondyloarthritis	Netherlands	Randomized controlled trial	Rheumatology—chronic lower back pain	Process change	Referral behavior	HCP factors	Referral rate Effectiveness
Full text	Keck, 2020	Primary care cluster RCT to increase diabetes prevention program referrals	USA	Other: randomized controlled trial and qualitative research	Diabetes	HCP education System change	HCP knowledge HCP attitudes and beliefs Referral behavior	HCP factors Service factors	Referral rate
Full text	Fonseca, 2018	Effectiveness of a referral program for early arthritis diagnosis at primary care centers in Portugal-final results from the SIARA study	Portugal	Randomized controlled trial	Rheumatoid arthritis (RA)	HCP education Process change System change	HCP knowledge HCP attitudes and beliefs Referral behavior	HCP factors	Referral rate
Full text	Mallett, 2014	Is physiotherapy self-referral with telephone triage viable, cost-effective, and beneficial to musculoskeletal outpatients in a primary care setting?	UK	Cohort study	Musculoskeletal/physiotherapy	Process change Patient intervention	Patient attitudes and beliefs	Patient factors	Referral rate Impact on existing service provision—improved waiting times, attendance rates Costs Patient satisfaction Patient experience process measures
Full text	McKay, 2019	Inter-professional integration improves the primary care to physical therapy referral process	USA	Non-randomized experimental study	Physiotherapy—musculoskeletal pain	System change	HCP knowledge HCP attitudes and beliefs Referral behavior Other: HCP collaboration	HCP factors Service factors	Referral rate Impact on existing service provision
Full text	Olayiwola, 2016	Electronic consultations to improve the primary care-specialty care interface for cardiology in the medically underserved: a cluster-randomized controlled trial	USA	Randomized controlled trial	Cardiology	Process change	HCP attitudes and beliefs Referral behavior	HCP factors Service factors	Referral rate Appropriateness of referral Staff satisfaction
Full text	Osteras, 2019	Implementing a structured model for osteoarthritis care in primary healthcare: a stepped-wedge cluster-randomized trial	Norway	Cluster-randomized controlled trial	Physiotherapy—Osteoarthritis	HCP education Process change System change	HCP knowledge HCP attitudes and beliefs Referral behavior HCP and patient interaction	HCP factors Patient factors Service factors	Referral rate Patient satisfaction
Full text	Perez, 2016	Understanding the causes of and developing effective interventions for schizophrenia and other psychoses	UK	Randomized controlled trial	Psychiatry	HCP education	HCP knowledge HCP attitudes and beliefs Referral behavior	HCP factors	Referral rate Referral quality Appropriateness of referral Costs
Full text	Pfeil 2023	A telemedicine strategy to reduce waiting lists and time to specialist care: a retrospective cohort study	Brazil	Retrospective cohort study	Mixed – nephrology Pulmonology Urology Neurology neuro- surgery rheumatology	Process change System change	Referral behavior	Service factors	Referral rate Impact on existing service provision Effectiveness Patient satisfaction
Full text	Quinlan, 2017	Implementing integrated care—lessons from the national implementation of general eReferrals in Ireland	Ireland	Qualitative research	All clinical specialties	Process change	Referral behavior	Service factors	Appropriateness of referral Impact on existing service provision
Full text	Rugkasa, 2020	Collaborative care for mental health: a qualitative study of the experiences of patients and health professionals	Norway	Qualitative research	Mental health	System change	HCP attitudes and beliefs Referral behavior HCP—patient interaction	HCP factors Patient factors Service factors	Referral quality Appropriateness of referral Impact on existing service provision Patient Satisfaction Staff satisfaction
Full text	Takashima, 2019	The impact of physician-directed versus patient-directed education on referral patterns to urogynecology	USA	Prospective, multi-phase, before-and-after study	Urogynecology	HCP education Patient intervention	HCP knowledge Referral behavior Patient knowledge	HCP factors Patient factors	Referral rate Referral quality

### Aims and evaluation of interventions

**Fig. 2:**
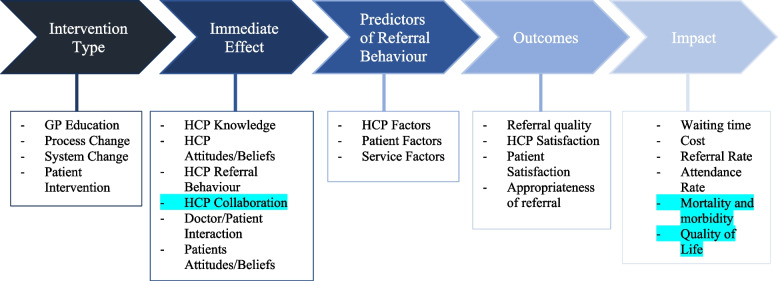
Logic model

In line with the Framework Synthesis approach [21], all included studies were organized into the framework developed in the original review [10] (Table 2). While the updated body of evidence broadly fit into the original framework, one new subheading “HCP collaboration” was identified under “immediate effect” (Fig. 2). Referral rate was an outcome in all studies included. As with the original review, appropriateness of referral, staff or patient satisfaction, cost implications, and impact on existing service provisions were all outcomes utilized to measure effectiveness. Additionally, under the heading “impact,” two new subheadings of outcome measures were identified: mortality and morbidity and quality of life (Fig. 2).

While all interventions were based in primary care, nine studies evaluated single-component interventions (e.g., a process change alone) [23–29], and thirteen were multi-component [30–44]. Eight interventions aimed to address only one effect factor, such as referral behavior, and ten were designed to affect multiple behaviors. Of the multi-component interventions, only two evaluated each component of the intervention [39, 45], making it challenging to assess which aspects had the most significant impact. Eight studies described the evaluation of their intervention as a clinical audit, pilot study, service evaluation, or feasibility trial [25, 28, 31, 34, 39, 40, 42, 45].

*GP* general practitioner, *HCP *health care professionals.

### Intervention development—rationale and theory for interventions

Seven studies cited a published protocol describing intervention development [26, 27, 35, 36, 38, 40, 43], three detailed the use of behavioral theory either in the protocol or main publication [35, 36, 43], and six outlined using an intervention framework [25, 26, 30, 35, 42, 43]. Applying the implementation of change in the healthcare framework [46] involved conducting a literature review and qualitative exploration with patients and HCPs to identify barriers and facilitators affecting the current care pathway prior to implementation [36]. Similarly, the application of the theory of planned behavior [47] enabled researchers first to understand the factors influencing the identification of patients who may require a referral. This was done using a questionnaire and semi-structured interviews with HCPs [36]. While Keck et al. did not directly use behavioral theory, their intervention was devised based on prior evidence from what is described as a landmark National Diabetes Prevention Program where it was applied [48]. Following the tailored interventions for chronic disease framework, authors conducted surveys and focus groups with HCPs and patients to determine what elements of the National Diabetes Prevention Program to include in their intervention.

Additional frameworks cited by the authors [25, 26, 42] include the Lean framework [49], the Framework for Scaling Health Interventions [50], and the Facilitated Process Improvement Approach [51]. Such approaches guided authors to conduct exploratory work as part of their development using either advisory group discussions or qualitative exploration [49–51].

While several studies did not include a theory or framework for intervention development, they reported development activities, such as collaboration with quality improvement teams and applying clinical guidance or available evidence [23, 27–29, 31, 37, 38, 40, 44]. Four studies did not report on intervention development [24, 33, 34, 44]; however, it is important to note that two of these studies were abstracts so may not have been able to provide that level of detail [33, 34].

### Mechanisms of referral

Eleven of the included studies did not specify the mode of referral, e.g., electronic, postal, or telephone.

 [27, 30–32, 35, 36, 38, 40, 41, 44, 52]. For these studies, while the aim was to improve the referral rate, the intervention did not include changing the mechanism of referral, and therefore, it was not mentioned. Of those that did specify, all detailed an electronic health system used for referral, multi-disciplinary communication, or electronic notifications [24–26, 28, 29, 33, 34, 37, 39, 42, 43].

### Intervention procedures or activities

**Fig. 3:**
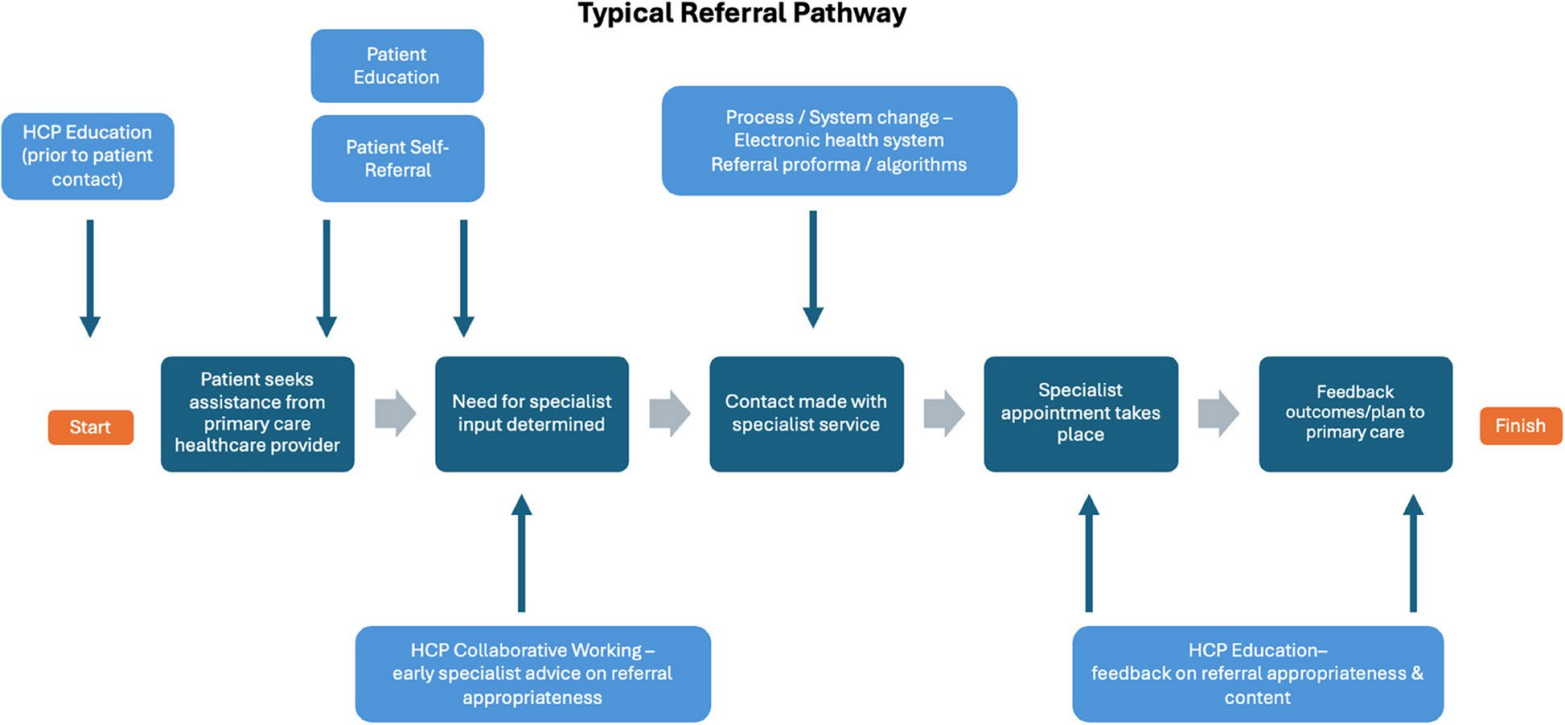
Typical referral pathway

Of the interventions evaluated, process [15] and system [10] changes were the most common. This included changes in the introduction of electronic health systems and referrals [24–26, 33, 39, 46], referral proformas and algorithms [24, 27, 42, 43], collaborative working such as having clinical specialists situated in primary care [29, 30, 33–35, 38, 41, 52], and patient direct access or self-referral [28, 40]. Figure 3 demonstrates the flow of a typical referral pathway and where such interventions might fit into this process, according to the studies evaluated.

### Process/system changes

Three studies implemented an electronic health system that enabled primary care staff and specialist clinicians to directly communicate with each other [24, 33, 52]. These systems enabled multi-disciplinary team co-management and peer-to-peer feedback on referral appropriateness [33]. All were described as an effective way to improve referrals. However, while introducing an e-referral system was acceptable to HCPs, in isolation, this did not always translate into an improvement in the time from referral to an appointment [34].

When considering referral proformas and algorithms, three studies included the introduction of an electronic system to flag patients who should be reviewed and considered for onward referral [37, 42, 43]. Referral proformas and algorithms implemented alongside HCP or process/system changes were found to be a successful way to improve referral rates, quality of referrals, and patient/staff satisfaction [24, 37, 42, 43]. However, the one study implementing a new referral algorithm alone was not effective at improving referral rates [27].

HCP collaborative working as a strategy to improve referral practice was implemented in different ways. One approach was the co-location of specialist clinicians embedded within primary care settings. Five studies [29, 38, 41, 43, 52] evaluated this approach positively seeing improvements in referral rates, high HCP satisfaction, and improvement in the flow of care delivery. Two studies [33] detailed the development of dedicated multi-disciplinary services, and one hired an additional medical assistant to identify patients who may need a referral prior to an appointment with a primary care professional [30]. Additionally, support staff roles, such as a “specialist champion,” were an effective way to share knowledge and create enthusiasm about the intervention amongst staff [43].

### HCP education

HCP educational programs were a common technique utilized in ten interventions, including tailored workshops, lectures, resources outlining referral criteria, and peer feedback [23, 31, 32, 35, 36, 41–44, 52]. Although most interventions included HCP education as an addition to a system or process change, in two studies, education programs were the only component [30, 36].

While the introduction of one virtual seminar for the multi-disciplinary team was well received by attendees, this single-component intervention did not improve referral rates [23].

In contrast, a theory-based educational intervention aiming to improve the identification and referral of adolescent patients with a high risk of first-episode psychosis included two face-to-face educational workshops with liaison practitioners, a pre-recorded video with additional information, and a postal campaign with written resources. Supporting staff beyond education, liaison practitioners were available to provide further guidance on a patient-by-patient basis. In this study, HCPs were receptive to the intervention and although the number of referrals and appropriateness improved, resulting in cost savings, it was not statistically significant. This can be seen in the higher rate of true-positive (appropriate) referrals in the intervention site (*p* = 0.02). Additionally, this also resulted in cost savings that were attributable to fewer false negative (inappropriate) referrals [36].

### Patient interventions

Interventions targeted at patients were the least common [3] and often coupled with HCP education and system or process changes. One study [19] included a brightly colored handout for physicians to use with their patients that outlined counseling on diet and exercise, possible treatment options, and space for the physician to write additional recommendations. A copy was then kept by physicians and patients and referred to when managing future care. This patient-centered intervention effectively improved dietician and aquatic therapy referrals for morbidly obese clinic patients [31].

A process change intervention included distributing information leaflets and posters in GP clinics to promote patient self-referral to physiotherapy. This successfully improved access rates and cost savings were observed [28]. However, patient-directed posters were not always successful, as an alternative study aiming to improve diagnosis numbers and referral rates saw no statistically significant differences compared to the control group [43].

### Barriers and facilitators to implementation

Six studies reported barriers and facilitators to the implementation of a referral improvement intervention [22, 25, 27, 26, 38, 41]. While the educational intervention detailed by Perez et al. improved liaison between primary and secondary care, authors reported that some GPs were unable to attend face-to-face workshops due to clinical priorities and time constraints. The use of the support provided by the specialist liaison practitioners was also low [31]; however, this was not seen in other studies included in this review where “site champions” were reported as an effective way to advance co-management goals and improve teamwork [42, 43]. Furthermore, the utilization of written resources sent to HCPs via email or post was found to be minimal [36, 43].

During the implementation of a diabetes prevention intervention, the perceptions of HCPs appeared to influence onward referral [43]. Some explained they were more likely to refer patients who they felt were motivated to improve their health and those with healthcare insurance. HCPs were also influenced by patients’ characteristics, such as literacy, age, and multimorbidity [43]. Referrals were less likely to be actioned in the presence of acute disease deterioration or those with chronic conditions, if patients had already initiated lifestyle changes [43]. Additionally, patient perceptions of the necessity of a specialist referral influenced referral practice. In those with a suspicion or at risk of dementia, 74% of patients who fit the referral criteria declined an appointment [41].

The co-location of GPs and specialist clinicians was identified as a factor of patient-centered case collaboration, with patients feeling that it enables GPs to be more involved in care. Through this strategy, HCPs praised the ability to quickly discuss patient’s cases, improving their confidence in referral appropriateness. However, barriers to this approach included logistical issues such as a lack of office space and inoperable funding structures [38].

Logistical issues were also reported in the evaluation of an e-referral platform. The authors report that respondents expressed policy uncertainty and worried that organizational structures and the current hospital administration may be unable to cope with wider implementation beyond the study [26].

## Discussion

The updated evidence in this literature highlights that single-component interventions alone may be less effective than those that use a multi-component approach. However, a published systematic review exploring the impact of multifaceted vs single-component healthcare interventions found no compelling evidence that multi-component interventions were more successful [53].

Reflecting on intervention components, as with the previous reviews, the co-location of primary care staff and specialist clinicians appears to improve referrals and patient experiences [36]. Similarly, while “site champions” were not utilized well by HCPs in all studies, there could be some benefit [36, 42, 43]. This is an approach that aligns well with the current NHS long-term plan, where a key aspect of the aim “supporting people to age well” includes bringing together different professionals to better coordinate care [54]. Although this approach may be successful in theory, given the staff shortages [55] and struggles with aging clinical facilities [56], it is questionable as to how this would be feasible to be implemented in most primary care NHS services across the UK.

Two studies also found self-referral strategies effective for MSK conditions [28, 40]. The ability for patients to self-refer is an increasingly popular strategy associated with increased referral appropriateness. Such practices are common and effectively utilized in UK mental health services, antenatal care, sexual health, and smoking cessation [57]. With its success, there is now the prospect of the approach being expanded to podiatry and incontinence assessments. Given the evidence in this review [28, 40], there could be scope for applicability in other clinical specialties providing services for patients with chronic conditions.

After process and system changes, healthcare education was the most common intervention component. The current evidence suggests that while it may not be effective in isolation, it is a useful way to improve referral rates and appropriateness. Interestingly, two studies highlighted a lack of uptake or use of written educational resources [36, 43]. However, as with the previous evidence, peer feedback improved referral time and appropriateness. While interventions in this review were mostly delivered in primary care, a solution to the poor utilization of educational resources could include harnessing the expertise of specialist clinicians. Supporting this, the British Medical Society has suggested approaches such as regular “interface groups” involving GP and secondary care representatives and outreach education led by specialist healthcare professionals as a useful way to improve referral practices and working relationships [58].

When considering barriers and facilitators to implementation, it is unsurprising that most face logistical issues and time constraints. However, it is particularly interesting that regardless of the financial accessibility of healthcare (fee-paying or free at the point of access), the perceptions of both healthcare professionals and patients appear to play a role in referral [12]. Despite there being qualitative evidence regarding barriers and facilitators to referral practices in different chronic conditions [59, 60], none of the studies included in this review specifically aimed to address or evaluate perceptions. This is work that should be undertaken prior to the development of any new interventions and to help guide such work, both intervention and behavioral frameworks should be utilized [15].

As the previous reviews did not explore intervention development or the use of frameworks and behavioral theory, it is difficult to ascertain if there has been an uptake in these types of methods. However, the studies that detail some form of systematic intervention development [25, 26, 30, 35, 42, 43, 49–51] appear to be effective and would be easier to replicate. This can be seen in the reporting of barriers and enablers, and of the six studies that did so, all included some form of systematic intervention development [22, 25, 27, 26, 38, 41]. The reporting of intervention development in this way allows readers to determine how they might go about employing a similar approach in their own clinical setting and if such barriers may apply in their practice.

### Study limitations

While this was a comprehensive review of the current literature, due to the heterogeneity of studies and differences in clinical context, it is difficult to compare intervention approaches or easily ascertain if they can be replicated. Additionally, of the seventeen full-text papers reviewed, nine achieved a score of less than 50% when appraised for quality. This suggests that while a relatively large body of evidence is available to guide clinical practice, we currently lack high-quality data, and this should be considered when applying such evidence to the development of future referral interventions.

Additionally, in this search for updated evidence, several studies were described by the authors as clinical audits, pilot studies, service evaluations, or feasibility trials, and only six out of the twenty-two publications evaluated were randomized control trials. While the exploration of clinical pathways is inevitably complex, and as such, a traditional RCT may not always be possible, if projects are not developed beyond the stage of pilot evaluation, it is difficult to see how an intervention may be implemented on a larger scale in organizations such as the NHS. Despite this, the increase in reporting intervention development methods is perhaps one of the most useful outcomes from this review as it allows for consideration of clinical applicability.

It is also important to highlight the decision to include abstract presentations within this review. Including abstracts in systematic reviews generates controversy as most are unable to sufficiently report the methodological approaches used. This makes it difficult to evaluate results and determine real-world applicability [61]. However, since the aims of this review were to identify components of interventions and mechanisms of referral where this information was present, it was included and considered.

While ten of the 23 studies took place in the UK and Europe, providing some insight into the implementation process in publicly funded healthcare systems, many of the studies evaluated were conducted in the USA (10/23). Since healthcare in the USA is largely a fee-paying service, this may impact how and when referrals are made and, in turn, affect the applicability of results in the NHS or European healthcare services. Despite this, it would largely be recognized that referrals to specialist services should occur in line with need and not in line with cost–benefit [62], and as such, when considering the results of this review, it is reasonable to assume the referrals are made in line with clinical guidance.

## Conclusion

In keeping with previous reviews [10, 11], a standard approach, in a one-size-fits-all manner, to improve referral practice is not associated with improved referral rates. Considering the updated literature against previous reviews, multi-component interventions addressing referral barriers in both primary and secondary care appear to be a successful way to improve referral rates, appropriateness, and staff satisfaction.

## Supplementary Information

Additional file 1.

Additional file 2: Quality Assessment.

## Data Availability

As this is a review article, all data included is publicly available. The datasets used and/or analyzed during the current study are available from the corresponding author upon reasonable request.

## Abbreviations

HCP: Healthcare professionals
GPs: General practitioners
NHS: National Health Service
TiDiER: Template for intervention description and replication
JBI: The Joanna Briggs Institute
