# Supplementary material for: Interventions to improve referrals from primary care to outpatient specialist services for chronic conditions: a systematic review and framework synthesis update
Source: Syst Rev. 2025 May 9;14:103. doi: 10.1186/s13643-025-02841-z (PMC12063302; doi:10.1186/s13643-025-02841-z)
Supplement: Supplementary file 1 — Additional file 1. [file 13643_2025_2841_MOESM1_ESM.docx]

**Appendix 1:**

**Search terms by group**

Refer OR Referral OR Consultations OR Community Referral

AND

General practic* OR GP OR Family practic* OR Nurs* OR Physiotherapist OR Podiatrist OR Heath care professional OR healthcare worker OR Self-refer OR Family Physician OR Family practitioner OR Doctor OR Physician OR Clinician

AND

Primary care OR Secondary care OR Specialist* adj4 care OR Specialist* adj4 service* OR Specialist* OR Specialisation OR Hospital* OR Clinic OR Outpatient OR Tertiary Health Care

AND

Intervention* OR Manag* adj4 demand

AND

Randomised controlled trial OR RCT OR Observational OR Cross-sectional OR Cohort OR Case control OR Qualitative

**Search terms applied in each database**

**Pro Quest**

tiab(Refer OR Referral OR Consultations OR ‘Community Referral’) AND tiab("General practic*" OR GP OR "Family practic*" OR Nurse* OR Physiotherapist OR Podiatrist OR "Heath care professional" OR "healthcare worker" OR "Self-refer" OR "Family Physician" OR "Family practitioner" OR Doctor OR Physician OR Clinician) AND tiab("Primary care" OR "Secondary care" OR Specialist* N4 care OR Specialist* N4 service* OR Specialist* OR Specialisation OR Hospital* OR Clinic OR Outpatient OR "Tertiary Health Care") AND tiab(Intervention* OR Manag* adj4 demand) AND tiab("Randomised controlled trial" OR RCT OR Observational OR "Cross-sectional" OR Cohort OR "Case control" OR Qualitative) AND (bdl(1007617 1008213) NOT at.exact("Commentary" OR "News" OR "Correspondence" OR "Letter to the Editor") AND la.exact("ENG") AND stype.exact("Scholarly Journals") AND pd(20131009-20231009) AND PEER(yes))

**Ovid:**

| 1 | Referral.ab,ti. |
| --- | --- |
| 2 | Consultations.ab,ti. |
| 3 | Community Referral.ab,ti. |
| 4 | "General practic*".ab,ti. |
| 5 | GP.ab,ti. |
| 6 | "Family practic*".ab,ti. |
| 7 | "Nurse*".ab,ti. |
| 8 | Physiotherapist.ab,ti. |
| 9 | Podiatrist.ab,ti. |
| 10 | Heath care professional.ab,ti. |
| 11 | healthcare worker.ab,ti. |
| 12 | Self-refer.ab,ti. |
| 13 | Family Physician.ab,ti. |
| 14 | Family practitioner.ab,ti. |
| 15 | Doctor.ab,ti. |
| 16 | Physician.ab,ti. |
| 17 | Clinician.ab,ti. |
| 18 | Primary care.ab,ti. |
| 19 | Secondary care.ab,ti. |
| 20 | (Specialist* adj4 care).ab,ti. |
| 21 | (Specialist* adj4 service*).ab,ti. |
| 22 | "Specialist*".ab,ti. |
| 23 | "Hospital*".ab,ti. |
| 24 | Clinic.ab,ti. |
| 25 | Outpatient.ab,ti. |
| 26 | Tertiary Health Care.ab,ti. |
| 27 | "Intervention*".ab,ti. |
| 28 | (Manag* adj4 demand).ab,ti. |
| 29 | Randomised controlled trial.ab,ot. |
| 30 | Randomised control trial.ab,ot. |
| 31 | Randomized controlled trial.ab,ot. |
| 32 | Randomized control trial.ab,ot. |
| 33 | RCT.ab,ot. |
| 34 | Observational.ab,ot. |
| 35 | Cross-sectional.ab,ot. |
| 36 | Cohort.ab,ot. |
| 37 | Case control.ab,ot. |
| 38 | Qualitative.ab,ot. |
| 39 | 1 or 2 or 3 or 4 |
| 40 | 5 or 6 or 7 or 8 or 9 or 10 or 11 or 12 or 13 or 14 or 15 or 16 or 17 or 18 |
| 41 | 19 or 20 or 21 or 22 or 23 or 24 or 25 or 26 or 27 |
| 42 | 28 or 29 |
| 43 | 30 or 31 or 32 or 33 or 34 or 35 or 36 or 37 or 38 or 39 |
| 44 | 40 and 41 and 42 and 43 and 44 |
| 45 | limit 45 to english language |
| 46 | limit 46 to yr="2013 - 2023" |

**Ebsco:**

| S1 | Refer OR Referral OR Consultations OR “Community Referral” |
| --- | --- |
| S2 | “General practic*” OR GP OR “Family practic*” OR Nurse* OR Physiotherapist OR Podiatrist OR “Heath care professional” OR “healthcare worker” OR “Self-refer” OR “Family Physician” OR “Family practitioner” OR Doctor OR Physician OR Clinician |
| S3 | “Primary care” OR “Secondary care” OR Specialist* N4 care OR Specialist* N4 service* OR Specialist* OR Specialisation OR Hospital* OR Clinic OR Outpatient OR “Tertiary Health Care” |
| S4 | Intervention* OR Manag* N4 demand |
| S5 | “Randomised controlled trial” OR RCT OR Observational OR “Cross-sectional” OR Cohort OR “Case control” OR Qualitative |
| S6 | S1 AND S2 AND S3 AND S4 AND S5 |
| S7 | S1 AND S2 AND S3 AND S4 AND S5 |
| S8 | S1 AND S2 AND S3 AND S4 AND S5 -  Limiters -  Publication Date: 20130101-20231231  Narrow by Language: - english  Search modes - Proximity |
