# Supplementary material for: Interventions to improve referrals from primary care to outpatient specialist services for chronic conditions: a systematic review and framework synthesis update
Source: Syst Rev. 2025 May 9;14:103. doi: 10.1186/s13643-025-02841-z (PMC12063302; doi:10.1186/s13643-025-02841-z)
Supplement: Supplementary file 2 — Additional file 2: Quality Assessment. [file 13643_2025_2841_MOESM2_ESM.docx]

**Table One**

| **Cohort Studies** | **Filippini** | **Barnett** | **Mallett** | **Pfeil** |
| --- | --- | --- | --- | --- |
| **Were the two groups similar and recruited from the same population?** | Yes | Unclear | Unclear | No |
| **Were the exposures measured similarly to assign people to both exposed and unexposed groups?** | Yes | Yes | Yes | Yes |
| **Were the exposures measured similarly to assign people to both exposed and unexposed groups?** | Unclear | Unclear | Yes | Unclear |
| **Were confounding factors identified?** | No | No | No | Yes |
| **Were strategies to deal with confounding factors stated?** | N/A | N/A | No | No |
| **Were the groups / participants free of the outcome at the start of the study (or at the moment of exposure)?** | Yes | Yes | Yes | Yes |
| **Were the outcomes measured in a valid and reliable way?** | Unclear | Unclear | Yes | Yes |
| **Was the follow up time reported and sufficient to be long enough for outcomes to occur?** | Yes | Yes | Yes | Yes |
| **Was follow up complete, and if not, were the reasons to loss to follow up described and explored?** | N/A | Yes | Yes | N/A |
| **Were strategies to address incomplete follow up utilized?** | N/A | Unclear | N/A | N/A |
| **Was appropriate statistical analysis used?** | Unclear | Unclear | Yes | Yes |
|  | 4/11 36% | 4/11  36% | 7/11  64% | 6/11  55% |

**Table Two Table Three**

| **Mixed Methods** | **Haley** | **Keck** |  | **Qualitative** | **Quinlan** | **Rugkasa** |
| --- | --- | --- | --- | --- | --- | --- |
| **Are there clear research questions?** | Yes | No |  | **Is there congruity between the stated philosophical perspective and the research methodology?** | No | No |
| **Do the collected data allow to address the research questions?** | Yes | Unclear |  | **Is there congruity between the research methodology and the research question or objectives?** | Yes | Yes |
| **Are the qualitative data collection methods adequate to address the research question?** | Unclear | Yes |  | **Is there congruity between the research methodology and the methods used to collect data?** | Yes | Yes |
| **Are the findings adequately derived from the data?** | Yes | Unclear |  | **Is there congruity between the research methodology and the representation and analysis of data?** | Yes | Yes |
| **Is the interpretation of results sufficiently substantiated by data?** | Unclear | No |  | **Is there congruity between the research methodology and the interpretation of results?** | Yes | Yes |
| **Is there coherence between qualitative data sources, collection, analysis and interpretation?** | Yes | No |  | **Is there a statement locating the researcher culturally or theoretically?** | No | Unclear |
| **Is the sampling strategy relevant to address the research question?** | Yes | N/A |  | **Is the influence of the researcher on the research, and vice- versa, addressed?** | No | No |
| **Is the sample representative of the target population?** | Unclear | N/A |  | **Are participants, and their voices, adequately represented?** | Yes | Yes |
| **Is the risk of nonresponse bias low?** | No | N/A |  | **Is the research ethical according to current criteria or, for recent studies, and is there evidence of ethical approval by an appropriate body?** | Yes | Yes |
| **Is the statistical analysis appropriate to answer the research question?** | Yes | N/A |  | **Do the conclusions drawn in the research report flow from the analysis, or interpretation, of the data?** | Yes | Yes |
| **Is randomization appropriately performed?** | N/A | No |  |  | 7/10  70% | 7/10  70% |
| **Are the groups comparable at baseline?** | N/A | No |  |  |  |  |
| **Are there complete outcome data?** | N/A | Yes |  |  |  |  |
| **Are outcome assessors blinded to the intervention provided?** | N/A | No |  |  |  |  |
| **Did the participants adhere to the assigned intervention?** | N/A | Yes |  |  |  |  |
|  | 6/15  40% | 3/15  20% |  |  |  |  |

**Table Four**

| **RCTs** | **Bishop** | **Jamal** | **Laires** | **Olayiwola** | **Osteras** | **Perez** |
| --- | --- | --- | --- | --- | --- | --- |
| **Was true randomization used for assignment of participants to treatment groups?** | Yes | Yes | Unclear | Unclear | Yes | Unclear |
| **Was allocation to treatment groups concealed?** | No | No | No | Yes | Yes | No |
| **Were treatment groups similar at the baseline?** | Yes | Yes | No | No | No | Unclear |
| **Were participants blind to treatment assignment?** | Unclear | No | No | No | No | Unclear |
| **Were those delivering the treatment blind to treatment assignment?** | No | No | No | Yes | No | No |
| **Were treatment groups treated identically other than the intervention of interest?** | Yes | Yes | Yes | Yes | Yes | Yes |
| **Were outcome assessors blind to treatment assignment?** | No | No | No | Yes | Yes | Unclear |
| **Were outcomes measured in the same way for treatment groups?** | Yes | Yes | Yes | Yes | Yes | Yes |
| **Were outcomes measured in a reliable way** | Yes | Unclear | Unclear | Yes | Yes | Unclear |
| **Was follow up complete and if not, were differences between groups in terms of their follow up adequately described and analysed?** | Yes | Unclear | Yes | Yes | Yes | Yes |
| **Were participants analysed in the groups to which they were randomized?** | Yes | Yes | Yes | Yes | Yes | Yes |
| **Was appropriate statistical analysis used?** | Yes | Yes | Yes | Unclear | Yes | Unclear |
| **Was the trial design appropriate and any deviations from the standard RCT design (individual randomization, parallel groups) accounted for in the conduct and analysis of the trial?** | Yes | Unclear | Yes | Unclear | Yes | Yes |
|  | 9/13  69% | 6/13  46% | 6/13  46% | 8/13  61% | 10/13  77% | 5/13  38% |

**Table Five Table Six Table Seven**

| **Case-Control** | **McKay** |  | **Quasi-Experimental** | **Takashima** |  | **Cross-Sectional** | **Disler** |
| --- | --- | --- | --- | --- | --- | --- | --- |
| **Were the groups comparable other than the presence of disease in cases or the absence of disease in controls?** | Unclear |  | **Is it clear in the study what is the ‘cause’ and what is the ‘effect’ (i.e. there is no confusion about which variable comes first)?** | Yes |  | **Were the criteria for inclusion in the sample clearly defined?** | Unclear |
| **Were cases and controls matched appropriately?** | Unclear |  | **Were the participants included in any comparisons similar?** | No |  | **Were the study subjects and the setting described in detail?** | Yes |
| **Were the same criteria used for identification of cases and controls?** | Yes |  | **Were the participants included in any comparisons receiving similar treatment/care, other than the exposure or intervention of interest?** | Unclear |  | **Was the exposure measured in a valid and reliable way?** | Unclear |
| **Was exposure measured in a standard, valid and reliable way?** | Yes |  | **Was there a control group?** | Yes |  | **Were objective, standard criteria used for measurement of the condition?** | Yes |
| **Was exposure measured in the same way for cases and controls?** | Yes |  | **Were there multiple measurements of the outcome both pre and post the intervention/exposure?** | No |  | **Were confounding factors identified?** | No |
| **Were confounding factors identified?** | No |  | **Was follow up complete and if not, were differences between groups in terms of their follow up adequately described and analyzed?** | Yes |  | **Were strategies to deal with confounding factors stated?** | No |
| **Were strategies to deal with confounding factors stated?** | N/A |  | **Were the outcomes of participants included in any comparisons measured in the same way?** | Yes |  | **Were the outcomes measured in a valid and reliable way?** | Unclear |
| **Were outcomes assessed in a standard, valid and reliable way for cases and controls?** | Yes |  | **Were outcomes measured in a reliable way?** | Yes |  | **Was appropriate statistical analysis used?** | Unclear |
| **Was the exposure period of interest long enough to be meaningful?** | Yes |  | **Was appropriate statistical analysis used?** | Yes |  |  | 2/8  25% |
| **Was appropriate statistical analysis used?** | Yes |  |  | 6/9  67% |  |  |  |
|  | 5/10  50% |  |  |  |  |  |  |
